# Preclinical PET and MR Evaluation of ^89^Zr- and ^68^Ga-Labeled Nanodiamonds in Mice over Different Time Scales

**DOI:** 10.3390/nano12244471

**Published:** 2022-12-16

**Authors:** Gordon Winter, Nina Eberhardt, Jessica Löffler, Marco Raabe, Md. Noor A. Alam, Li Hao, Alireza Abaei, Hendrik Herrmann, Claudia Kuntner, Gerhard Glatting, Christoph Solbach, Fedor Jelezko, Tanja Weil, Ambros J. Beer, Volker Rasche

**Affiliations:** 1 Department of Nuclear Medicine, Ulm University Medical Center, 89081 Ulm, Germany; 2 Department of Internal Medicine II, Experimental Cardiovascular Imaging, Ulm University Medical Center, 89081 Ulm, Germany; 3 Department of Synthesis of Macromolecules, Max Planck Institute for Polymer Research, 55128 Mainz, Germany; 4 Department of Biomedical Imaging and Image-Guided Therapy, Medical University Vienna, 1090 Vienna, Austria; 5 Institute for Quantum Optics, Ulm University, 89081 Ulm, Germany

**Keywords:** nanodiamonds, PET imaging, MRI imaging, biodistribution, nanoparticles, ^89^Zr, preclinical, SCID mice, C57BL/6

## Abstract

Nanodiamonds (NDs) have high potential as a drug carrier and in combination with nitrogen vacancies (NV centers) for highly sensitive MR-imaging after hyperpolarization. However, little remains known about their physiological properties in vivo. PET imaging allows further evaluation due to its quantitative properties and high sensitivity. Thus, we aimed to create a preclinical platform for PET and MR evaluation of surface-modified NDs by radiolabeling with both short- and long-lived radiotracers. Serum albumin coated NDs, functionalized with PEG groups and the chelator deferoxamine, were labeled either with zirconium-89 or gallium-68. Their biodistribution was assessed in two different mouse strains. PET scans were performed at various time points up to 7 d after i.v. injection. Anatomical correlation was provided by additional MRI in a subset of animals. PET results were validated by ex vivo quantification of the excised organs using a gamma counter. Radiolabeled NDs accumulated rapidly in the liver and spleen with a slight increase over time, while rapid washout from the blood pool was observed. Significant differences between the investigated radionuclides were only observed for the spleen (1 h). In summary, we successfully created a preclinical PET and MR imaging platform for the evaluation of the biodistribution of NDs over different time scales.

## 1. Introduction

The interest in labeled nanoparticles (NPs) has steadily increased in recent years, as they are attractive for use as drug carriers or for imaging. Nanodiamonds (NDs) are a class of nanoparticles with the advantage of their carbon-based and chemically inert composition. These particles are also of specific interest for MR imaging with regard to the possibility of hyperpolarization [1,2,3,4,5,6,7]. Several research groups are working to establish the hyperpolarization of irradiated carbon-13 enriched NDs as highly sensitive MRI contrast agent. However, for a general assessment of the pharmacokinetic features of NDs, PET imaging appears attractive due to its excellent sensitivity, quantitative properties, and long-term monitoring capabilities [8,9,10,11,12,13]. Positron emission tomography (PET) using long-lived radionuclides allows for non-invasively quantifying blood retention, biodistribution, and potential unspecific accumulation in the filter organs of the reticuloendothelial system (RES). While studies on the radiolabeling of NPs with fluorine-18 (^18^F), copper-64 (^64^Cu), zirconium-89 (^89^Zr), germanium-69 (^69^Ge), technetium-99 m (^99m^Tc), or iodine-radioisotopes as well as pretargeted approaches for PET and single photon emission computed tomography (SPECT) have been investigated by different groups, only a few biodistribution or tumor accumulation analyses of NDs have been published so far [14,15,16,17,18,19,20,21,22,23,24,25]. Therefore, we focused on creating a preclinical PET and MR imaging platform to evaluate and monitor the distribution and accumulation of NDs over different time scales. For this purpose, we performed preclinical in vivo PET imaging biodistribution studies of radiolabeled NDs with human serum albumin (HSA) coating in both immunocompetent and immunodeficient mice strains using both short- and long-lived radionuclides. 

The radionuclide gallium-68 (^68^Ga) with a short half-life of t_1/2_ = 67.7 min is an attractive isotope for PET measurements. It is widely used as ^68^Ge/^68^Ga generators are available in many facilities [26]. The biodistribution of ^68^Ga-labeled substances can be readily observed over short periods of time [27,28]. Measurements over longer times are facilitated using zirconium-89 (t_1/2_ = 78.4 h) [29,30,31]. This radionuclide is also commercially available. Both nuclides can be coordinated with the chelating agent deferoxamine [32].

HSA is the most abundant protein in blood plasma (blood concentration of 35–50 g/L) and will interact with injected NDs. Coating with HSA is expected to prevent immunogenic interactions. Studies with HSA-binding or coating have demonstrated promising effects, such as reducing toxicity and increasing drug availability [33,34,35,36,37,38,39].

Since mouse strain dependent organ variation has been reported [40], the suggested approach was tested in two of the most commonly used wildtype (WT, C57BL/6) and xenograft generating (SCID) mouse strains [41,42,43]. Where the wild-type strain is well qualified for biodistribution analysis, the SCID mice provide an excellent model for tumor xenograft generation and metastasis studies [41]. Even though no specific tumor targeting was addressed in this work, tumor models are of interest as nanoparticles tend to accumulate in solid tumors due to the EPR effect [44,45,46,47,48]. In this work, the SCID model was preferred to the NOD or NOD/SCID model, in which the macrophage development and function is disrupted [43], causing reduced accumulation in liver, lung, or spleen. For all experiments, nanodiamonds with HSA-coating and a specific chelator (DFO) for radionuclide binding were used. The obtained results in wildtype and immunodeficient mice clearly indicate the potential use of the suggested approach for longitudinal monitoring of the nanodiamonds after systemic i.v. injection.

The results of those studies can serve as a reference for future distribution studies of further refined NDs, e.g., functionalized NDs for specific target structures as well as for hyperpolarized NDs in MR imaging experiments.

## 2. Materials and Methods

### 2.1. Preparation and Coating of the Nanodiamonds (NDs)

NDs (MD100) with a diameter of around 100 nm (based on transmission electron microscopy, Figure S2A) were used. The carbon-13 enriched NDs were provided by Microdiamant AG (Lengwil, Switzerland) and irradiated with 10 MeV electrons from a linear electron accelerator (MB10–30MP; Mevex Corp., Stittsville, ON, Canada) at the IOM in Leipzig, according to Laube et al. [49]. The NDs were coated with an albumin protein which was modified with *p*-SCN-Bn-deferoxamine (DFO, 752 g/mol; Macrocyclics, Inc., Plano, TX, USA) groups enabling zirconium-89 and gallium-68 labeling of the coated NDs.

The coating is based on human serum albumin (HSA) as reported previously [34]. In brief, the HSA (Sigma-Aldrich, St. Louis, MO, USA) was cationized and PEGylated (cHSA-PEG). The carboxylic acid groups of HSA were converted into primary amino groups by reaction of HSA with ethylenediamine, thereby increasing its molecular weight from 66 kDa (native HSA) to 72 kDa (cHSA, Figure S1). Next, about 16 PEG (Rapp Polymere GmbH, Tübingen, Germany) chains with a molecular weight of 2000 g/mol were attached to the cationized HSA as indicated by the further increase in molecular weight of 33 kDa yielding cHSA-PEG. Subsequently DFO-isothiocyanate was coupled by reacting the isothiocyanate with the primary amine groups of the cHSA-PEG. Hence, 50 equivalents of DFO were added to cHSA-PEG in phosphate buffer (50 mM, pH 8) and the mixture was stirred overnight. Unreacted DFO was removed by ultrafiltration (Vivaspin 20, molecular weight cut-off 30 kDa, Sartorius AG, Goettingen, Germany). A further increase in mass by 11 kDa indicated the coupling of roughly 15 DFO units on average yielding the final coating cHSA-DFO.

For coating of the NDs with the cHSA-DFO, the NDs were first diluted to low concentration (0.1 mg/mL) in boric acid buffer (50 mM, pH 8.4; Sigma-Aldrich, St. Louis, MO, USA). Afterwards, a solution of the cHSA-DFO (0.1 mg/mL) in boric acid (50 mM, pH 8.4) was added to the NDs suspension (mass ratio: 4:1 cHSA-DFO:NDs) and the mixture was stirred overnight. Free proteins were removed by centrifugation (18,000× *g*, 20 min), and successful coating was confirmed by dynamic light scattering (DLS, Zetasizer Nano-S90 (Nano series); Malvern Panalytical, Malvern, Worcestershire, UK). The averaged diameter of the coated nanodiamonds increased from 130.8 ± 2.9 nm (PDI: 0.086) to 170.1 ± 0.9 nm (PDI: 0.133, Figure S2B) in DLS.

### 2.2. Radiolabeling and Stability Testing

Zirconium-89 (half-life *t*_1/2_ = 78.4 h) was produced as reported previously [29], with some further optimizations. In brief, after dissolving the irradiated yttrium (Alfa Aesar, Haverhill, MA, USA; 99.9 %) in 6 M HCl (diluted from concentrated acid, 2 mL (Merck KGaA, Darmstadt, Germany)) and adding 2 M HCl (2 mL), the solution was transferred to a hydroxamate resin cartridge (TrisKem, Bruz, France, ZR Resin, 2 mL). The cartridge was rinsed with 2 M HCl (10 mL) and H_2_O (10 mL; VWR International, Radnor, PA, USA, Normapur grade). Extraction was performed with 0.5 M oxalic acid (Sigma Aldrich, St. Louis, MO, USA, 5 mL) and the eluate was transferred to an anion exchange cartridge (*Sep*-*Pak* Accell Plus *QMA* Plus *Light*, Waters GmbH, Eschborn, Germany). The anion exchange cartridge was rinsed with 40 mL H_2_O (Normapur grade) and dried. Elution was achieved by the addition of 2 × 500 µL of 1 M HCl.

Gallium-68 (*t*_1/2_ = 67.7 min) was produced using a ^68^Ge/^68^Ga generator (iThemba LABS, Johannesburg, South Africa). The generator was eluted with 0.6 M HCl and small fractions were collected to achieve high concentrations of the ^68^Ga nuclide.

As preparation, the nanodiamonds were stored in water at a concentration of 500 µg/mL at 4 °C and vortexed before being dispersed with ultrasound at room temperature for about 30 to 45 min. For radiolabeling, 10 MBq of [^89^Zr]ZrCl_4_ were adjusted to a pH value of 5 to 6 with a 1 M Na_2_CO_3_ (Merck KGaA, Darmstadt, Germany) solution. 0.9 % NaCl (B. Braun SE, Melsungen, Germany) was added to the batches to ensure similar total volumes of 600 μL to 650 µL. The activity was transferred into prepared BSA (bovine serum albumin, Sigma-Aldrich, St. Louis, MO, USA) coated (5 % BSA in phosphate-buffered saline (PAN-Biotech GmbH, Aidenbach, Germany) for 1 h) “low-bind protein” tubes (Eppendorf SE, Hamburg, Germany). At a capacity of the chelating agent DFO of 3.4 % (*w*/*w*), an ND amount that resulted in an excess of DFO relative to the radionuclide was used for optimal radiolabeling: 1500-fold DFO for zirconium-89 and up to 92,000-fold for gallium-68 labeled NDs. The samples were incubated at room temperature for 30–60 min (gallium-68) or 60–120 min (zirconium-89) in an overhead shaker (Multi Bio RS-24, Biosan, Riga, Lettland). At various times, the radiolabeling of the NDs was monitored by thin layer chromatography (TLC; Silica gel 60 RP-18 F_254_s sheets (Merck KGaA, Darmstadt, Germany); 0.1 M citrate buffer pH 5.0). The TLCs were evaluated using a phosphorimager (FLA3000, Fujifilm, Tokyo, Japan) and the relative content of free radionuclide and labeled compound was determined in percent.

For stability testing, samples with ^89^Zr-labeled NDs were centrifuged for 30 min at 10,000 rpm at 4 °C (Mikro 220R, Andreas Hettich GmbH & Co. KG, Tuttlingen, Germany). The supernatants were discarded, and precipitates of the NDs were individually resuspended with equal amounts of either 0.9% NaCl, human serum, or cell culture medium (of the LNCaP C4-2 cell line). The preparations were incubated at room temperature in an overhead shaker and the stability of the ^89^Zr-labeling on the NDs was tested by thin-layer chromatography at different time points of up to six days. The evaluation of the TLCs was performed with a phosphorimager and the corresponding analysis software (AIDA image analyzer, ver. 4.24, (Elysia-Raytest GmbH, Straubenhardt, Germany).). An overview illustration of the radiolabeling process can be found in the supplement (Figure S3).

### 2.3. Cell Culture

The androgen-independent prostate carcinoma (PCa) cell line LNCaP C4-2 (ViroMed Laboratories, Minnetonka, MN, USA) [50] and the PSMA-negative PCa control PC-3 (ACC465, DSMZ, Braunschweig, Germany) [51] were used to establish xenografts in the mouse model. The cell line LNCaP C4-2 is known to form highly vascularized tumors [52] while PC-3 tumors are highly proliferating and grow more invasive [53]. The tumor cell lines are particularly suitable for uptake studies with regard to the EPR effect due to their good vascularity. In addition, the xenograft models can be used for future studies using NDs with PSMA-specific ligands. The cell lines were cultivated as described elsewhere [54]. Cells were counted using a Neubauer improved hemocytometer (C-Chip, DHC-N01, NanoEnTek, Seoul, Republic of Korea).

### 2.4. Mouse Models

The biodistribution of radioactively labeled NDs was studied in male C57BL/6NCrl mice (C57BL/6, n = 10; Charles River Laboratories, Sulzfeld, Germany) and in male immunodeficient CB17/lcr-Prkdc scid/Crl mice (SCID; n = 33; Charles River Laboratories, Sulzfeld, Germany). Tumor xenografts of the human prostate carcinoma cell lines LNCaP C4-2 and PC-3 were established by the administration of 1 × 10^6^ cells subcutaneously into the subscapular regions (left = LNCaP C4-2; right = PC-3) in a subset of the SCID mice (n = 15) (Table 1). Tumor cells were applied with one week difference and tumors grew in a period of 3 weeks after injection of LNCaP C4-2 and two weeks for PC-3, respectively. 

The studies were approved (ethical approval code 1375) by the national authority (Regierungspräsidium Tübingen, Baden-Württemberg) in compliance with German laboratory animal experimentation act and study procedures were in accordance with the European Communities Council Directive of 22 September 2010 (2010/63/EU). All applicable institutional and national guidelines for the care and use of animals were followed.

### 2.5. In Vivo Measurements

PET and MRI data were obtained for biodistribution (PET) assessment after systemic injection of the NDs and anatomical reference (MRI). The data were spatially co-registered and the PET biodistribution data superimposed onto the MRI anatomic data for proper anatomic identification of the origin of the radiation.

For scanning, animals were continuously anesthetized with isoflurane (2.5% for initiation, 1.5% during preparation and measurements) in an oxygen/compressed air mixture (20/80 %/%). Between the subsequent PET measurements, the mice could move freely in the cage, while between MR and PET measurement the animal was held anesthetized to prevent movement and allow for superposition of the data. An overview illustration of the in vivo measurements can be found in Figure S4.

#### 2.5.1. MR Measurements

For anatomical reference, high-resolution anatomical images were obtained with an 11.7T BioSpec 117/16 MRI/MRS (Bruker, Ettlingen, Germany) dedicated small animal system operating on ParaVision 6.1. To minimize overall anesthesia time, a fast multi-slice gradient echo sequence with acquisition parameters as: echo/repetition time TE/TR = 1.5 ms/150 ms, spatial resolution ∆r = 100 × 140 × 1000 μm^3^, flip angle α = 15°, and NSA = 12 signal averages was used. During scanning, animal respiratory rate was constantly monitored (SA Instruments Inc., Stony Brook, NY, USA) and the isoflurane level adjusted (1–1.5% in pressured air) to achieve a constant respiratory rate between 60 and 80 cycles per minute.

#### 2.5.2. PET Measurements

Following the intravenous injection of NDs into the tail vein, up to five time points, depending on the radionuclide used (1 h, 3 h, 24 h, 3 d and 7 d), were investigated with 4–7 animals each. Dynamic PET scans (Focus120, Siemens Medical Solutions, Inc., Erlangen, Germany) were performed over 60 min (first measurement including injection, 1 h) and 30–60 min (3 h, 24 h, 3 d, 7 d) with animals positioned on a heat mat with the scapular region in the central field-of-view. For PET scans an energy window of 350–650 keV and a timing window of 6 ns was used. Subsequently, a 515 s attenuation correction scan using a cobalt-57 point source was performed. Based on the list-mode data of the emission scans, histograms were created for a single 30- or 60-min image and additional 6 or 12 dynamic frames of 300 s each. In the case of catheter injection, during the first measurement, 6 × 20 s, 7 × 60 s, and 10 × 300 s frames were used for histograming. Images were reconstructed using the OSEM3D/MAP algorithm which consists of 4 OSEM2D, 2 OSEM3D and 18 MAP iterations. In addition to the main SCID-mice model with injection of [^89^Zr]Zr-DFO-NDs, two models with minor number of animals ([^89^Zr]Zr-DFO-ND in C57BL/6 mice and [^68^Ga]Ga-DFO-ND in SCID mice) were analyzed in parallel for comparison (Table 1). A selection of the animals was measured repeatedly at various time points for continuous datasets.

### 2.6. Gamma Counter Measurements

For validation of the PET-derived biodistribution, the investigated animals (n = 4) were sacrificed at each time point. Organs and tissue samples (blood, heart, brain, lung, liver, spleen, kidney, colon, small intestine, muscle, bone and tail, tumor if available) were extracted for further individual quantification of the organ-specific ND accumulation by means of gamma counting (COBRA II, Perkin Elmer, Waltham, MA, USA). The organs and tissue samples were weighed separately, and the gamma counter data were decay corrected to the time of the ND injection. The data were corrected by the injected activity (IA) and calculated as percent injected activity (%IA) or percent injected activity per gram tissue (%IA/g). For the comparison of the models, the respective organ weight was normalized to the weight of the corresponding mouse to minimize differences due to different body weight. 

### 2.7. Data Evaluation and Statistics

For the evaluation of biodistribution, a volume of interest (VOI) was identified manually based on PET and MR images for liver, spleen, heart or mediastinum, brain, and, if available, tumors. The respective time activity curves (TAC) were generated over according to the respective histograming. All time points were corrected to the specific injection time. Additionally, values in (%IA/mL) were selected from the last 5 min of dynamic PET measurements for analysis to allow accurate comparison with the gamma counter data.

Image data were superimposed and evaluated using the following software tools: AsiProVM (Siemens Medical Solutions, Inc., Erlangen, Germany), Vinci64 (Max-Planck-Institut für Stoffwechselforschung Köln, Germany) [55], 3DSlicer 4.11 (www.slicer.org (accessed on 16.12.2022) [56] and PMOD 4.106 (PMOD Technologies LLC, Zurich, Switzerland, www.pmod.com/web/ (accessed on 16.Dec.2022)). MS Excel 2019 (Microsoft) was used to analyze the quantification measurements. Outlier detection (ROUT-test, Q = 1 %), statistical evaluation (unpaired t-test (Mann–Whitney)), one-phase association, and non-linear regression for curve fitting (to a tri-exponential function [57]) were performed using GraphPad Prism version 9.4.0 for windows (GraphPad Software, San Diego, CA, USA, www.graphpad.com (accessed on 16.12.2022)). In detail, organ weights were compared using an unpaired t-test, including test for normality. Blood correlation analyses were performed using SPSS (IBM Corp. Released 2021. IBM SPSS Statistics for Windows, Version 28.0.1.0 Armonk, NY, USA: IBM Corp). Pearson correlation coefficients were determined for ^89^Zr-labeled NDs in SCID mice (I) based on gamma counter data.

## 3. Results

### 3.1. Radiolabeling of DFO-NDs and Stability of Radiolabeled DFO-NDs

**Figure 1:**
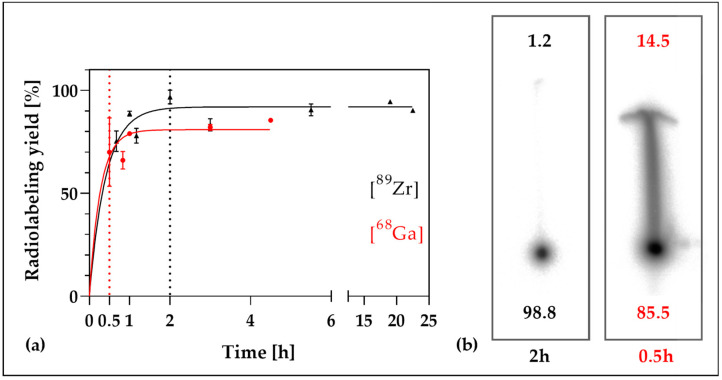
Radiolabeling of the nanodiamonds. ^89^Zr- and ^68^Ga-labeling diagram of bound activity in terms of time, followed for 4.5 h (^68^Ga) and 24 h (^89^Zr), respectively (**a**). For zirconium-89 already after 2 h of incubation (97 ± 3)% of the radioactivity was bound to the nanodiamonds (**a**) as observed by thin layer autoradiography. In the exemplary TLC the relative proportion of bound and free zirconium-89 or gallium-68 is given in percent (**b**).

**Figure 2:**
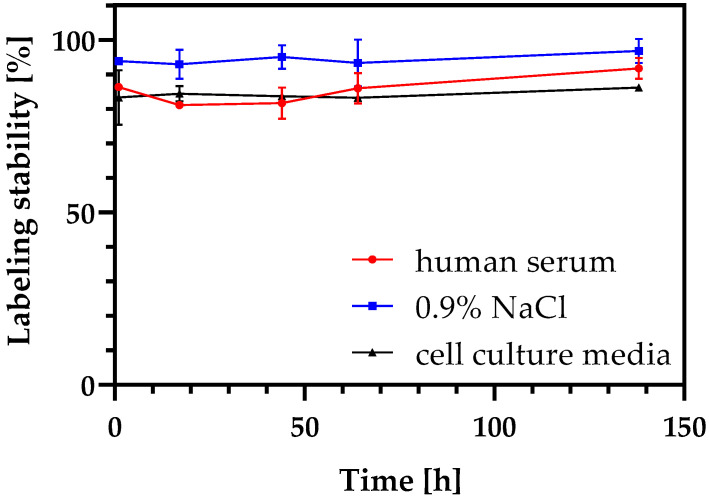
^89^Zr-labeling stability in human serum, 0.9% NaCl and cell culture media. Over the entire measuring period of 138 h, the labeling remained stable in all samples.

The radiolabeling of the NDs, either with zirconium-89 or gallium-68 was successfully accomplished. For [^89^Zr]Zr-DFO-ND, already after 120 min, approximately (97 ± 3)% of the radionuclide was bound, while for [^68^Ga]Ga-DFO-ND after 30 min (70 ± 16) % of the activity was bound (Figure 1). The labeling remained verifiably stable over a period of 138 h under the conditions tested (Figure 2). According to available comparative studies, serum stability tests for [^68^Ga]Ga-DFO-ND can be expected to give the same results [32,58].

### 3.2. Comparison of the Organ Weights

**Figure 3:**
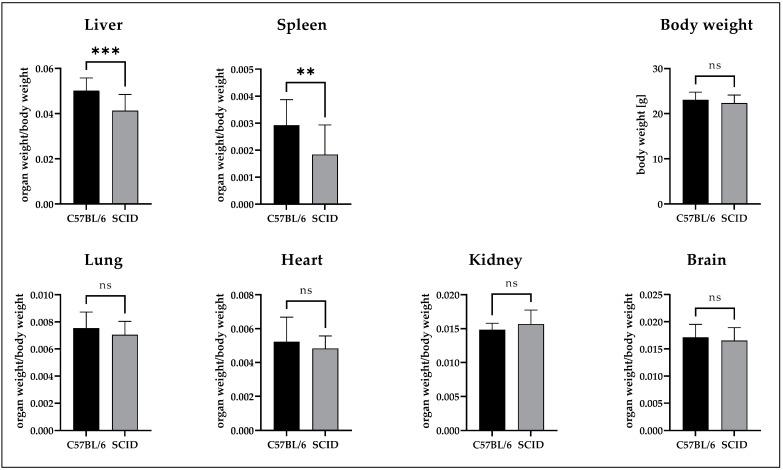
Comparison of the weights of various relevant organs in the mouse models C57BL/6 and SCID. For the mice models equal average body weights were observed. The organ weights were normalized to the respective individual body weight. A significant difference was detected for liver and spleen, while for lung, heart, kidney and brain no relevant differences could be observed. ** *p* < 0.005; *** *p* < 0.0005; ns = not significant.

The weight of the excised organs is shown in Figure 3 for the C57BL/6NCrl mice (n = 10) and the immunodeficient CB17/lcr-*Prkdc*^scid^/IcrIcoCrl mice (n = 33). An unpaired non-parametric test (Mann–Whitney) revealed a significant difference in the organ weight of liver (1.2× smaller in SCID) and spleen (1.8× smaller in SCID), while lung, brain, kidney, and heart weight did not show any significant differences. The average body weight was nearly identical with 22.3 ± 1.9 g (SCID) and 23.1 ± 1.7 g (C57BL/6).

### 3.3. Evaluation of PET and MR-Based Imaging

**Figure 4:**
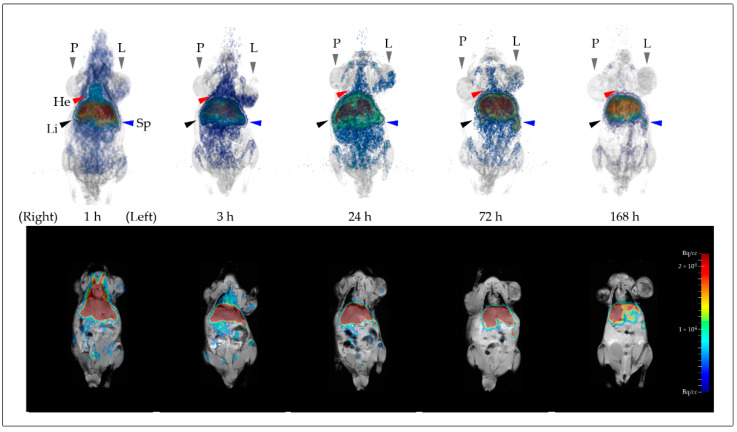
Fused PET and MR imaging data for a single SCID-mouse injected with [^89^Zr]Zr-DFO-NDs (I) measured 1 h, 3 h, 24 h, 3 d and 7 d post injection. In the anterior view a maximum intensity projection (MIP) is combined with a volumetric representation of the MR data (upper row). Additionally, a coronal MR section is combined also in the anterior view, with the respective 2D PET images. The PET data were well matched to the specific tissues by the anatomical image information from MRI. The labeled organs are He = Heart, Li = Liver, Sp = Spleen, P = PC-3 tumor, L = LNCaP C4-2 tumor.

PET and MR scans could be successfully performed after injection of [^89^Zr]Zr-FDO-NDs in all respective animals and for all time points (1 h, 3 h, 24 h, 3 d, and 7 d post injection), as depicted in Figure 4, with PET data superimposed onto the MRI anatomic images.

A clear accumulation of the NDs at 1 h p.i. was observed by visual qualitative analysis in the heart region/blood pool (i.e., the blood-filled heart chambers), the liver and the spleen. The distribution throughout the body decreased over the measurement period, as did the signal in the cardiac region, both correlated to the blood pool. For liver and spleen, only a minor decrease in signal was observed during the observation period. At 3 h, 24 h, and 72 h, a signal was also detected in the LNCaP C4-2 tumor. 

Due to the short half-life, PET measurements for gallium-68 labeled nanodiamonds were restricted to 1 h and 3 h (Figure S5). A similar primary accumulation in the liver and spleen was observed, where tumors did not show clear enhancement within this frame.

The rapid accumulation in liver and spleen is evident and remains stable throughout the measurement period. Accumulation in the cardiac region (He) can be observed at 1 h p.i. The signal decreases distinctly in the duration of the measurement. Moreover, note the accumulation in the LNCaP C4-2 tumor region (L) starting at 1 h and decreasing from 24 h to 168 h.

### 3.4. Ex Vivo Biodistribution Validation Based on Gamma-Counter Data

**Figure 5:**
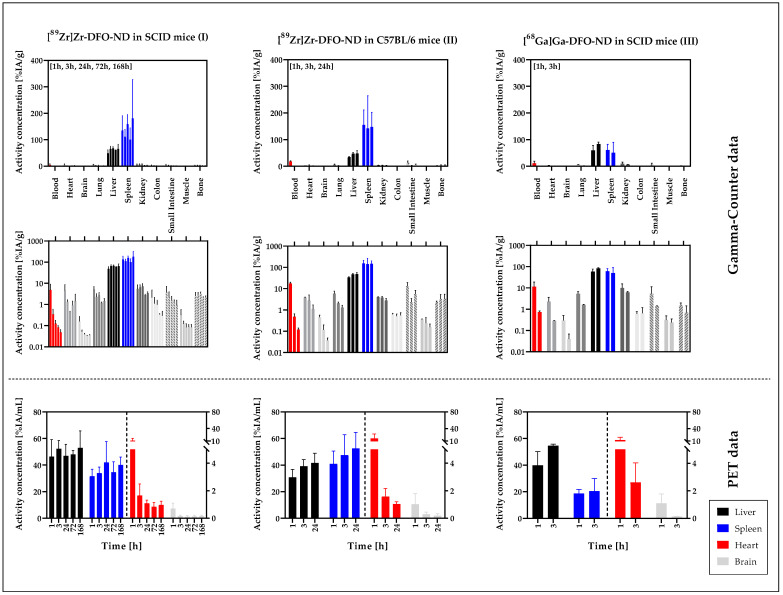
Mean and SD of weight-normalized organ accumulation [%IA/g] of radiolabeled NDs in the respective mouse model based on gamma counter evaluation and compared with the PET-based evaluation. The gamma counter-based evaluation is presented in the top row (linear scale) and middle row (logarithmic scale), whereas the PET-based data are presented in the bottom row (linear scale). For PET data, liver and spleen values are associated with the left ordinate, while heart and brain values are associated with the right ordinate. Liver, heart (blood, in case of gamma counter data), and spleen as the main organs of activity distribution are highlighted in color.

The mean accumulation for each organ was calculated as a percent of the injected activity with respect to organ weight (%IA/g) (Figure 5). The values are summarized in Tables S1–S3.

In good consistency with the image data, the major accumulation of NDs was detected in the liver and spleen with maximum activity concentrations in liver (mean ± SD): (67.1 ± 7.7) %IA/g (I, 24 h), (48.1 ± 10.8) %IA/g (II, 24 h), (84.0 ± 6.6) %IA/g (III, 3 h) and in spleen (180.4 ± 148.0) %IA/g (I, 168 h), (155.0 ± 56.5) %IA/g (II, 1 h), and (60.8 ± 21.3) %IA/g (III, 1 h). For the ^89^Zr-labeled NDs, a higher activity concentration was detected in the spleen than in the liver, whereas similar activities were detected for the ^68^Ga-labeled NDs in these organs. In the liver, a slight increase in signal was detected up to 24 h p.i., after which the signal remained constant.

For all three study models analyzed (Table 1), in blood, the detectable signal decreased significantly over time by up to two orders of magnitude (I: (4.8 ± 4.3) %IA/g (1 h); (0.05 ± 0.02) %IA/g (168 h)). A similar decreasing trend was observed for brain, heart, lung, small intestine, colon, and muscle. For kidney and bone, no significant signal variations could be detected over time.

In all three study models, the highest accumulation was observed in the liver and spleen and in the blood pool at the first measurement. With the gamma counter, accumulation in the spleen appears to be higher than in the liver in models I and II, while similar accumulation was observed in the liver and spleen ex vivo in model III ([^68^Ga]Ga-DFO-ND in SCID mice). In PET measurements, the difference between liver and spleen was minimal in study model II, whereas in models I and III accumulation was higher in liver than in spleen.

**Figure 6:**
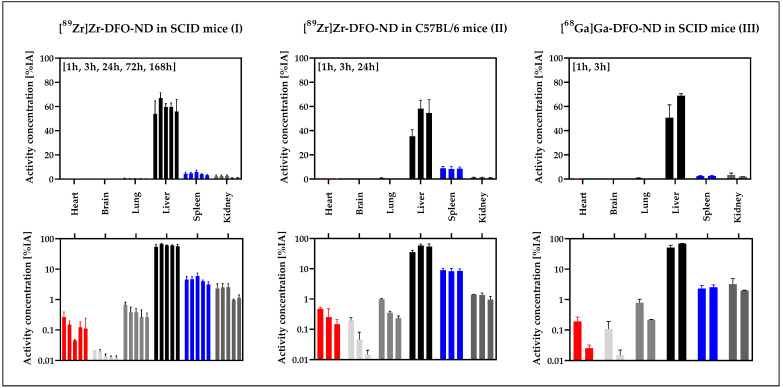
Biodistribution of radiolabeled NDs in %IA (mean and SD) for the various models. The gamma counter-based evaluation is presented in linear scale (upper row) and logarithmic scale (lower row). Only fully excised organs were included for evaluation in %IA. Of the total applied activity (100%) about 60% was accumulated in the liver (black) over time, while less than 10% was detected in the spleen (blue), kidney and lung.

For all organs completely excised, including heart, brain, lung, liver, spleen, and kidney, the percentage of the injected activity (%IA) was quantified (Figure 6) for the organ-specific assessment of the ND uptake.

It was clearly demonstrated that the majority of the applied NDs accumulated in the liver: (66.9 ± 4.4) %IA (I, 3 h), (58.2 ± 7.0) %IA (II, 3 h), (68.8 ± 1.7) %IA (III, 3 h). Less than 10% accumulated in the spleen (6.0 ± 1.4) %IA (I, 24 h), (8.9 ± 1.5) %IA (II, 1 h), (2.6 ± 0.5) %IA (III, 3 h). After a slight increase up to 3 h, the signal in the liver decreased only marginally at later measurement time points. The signals in spleen and kidney also remained at a constant level after the first hour. For heart, brain, and lung, a signal decrease over time was observed. The profile and magnitude of activity accumulation over time is similar for all three models.

To analyze significant differences between the three models, gamma counter data from liver and spleen at 1 h, 3 h, and 24 h, based on %IA/g and %IA calculations were compared (Figures S6 and S7). Significant differences were found between ^89^Zr-labeled NDs in SCID mice (I) and ^89^Zr-labeled NDs in C57BL/6 mice (II) at 3 h and 24 h for the liver data (*p* < 0.05, Figure S6). For the spleen, a significant difference was observed between ^89^Zr-labeled NDs in SCID mice (I) and ^68^Ga-labeled NDs in SCID mice (III) at 1 h.

After normalization to organ weight (%IA), no significant difference was detected between liver data (Figure S7). For the spleen, a significant difference (*p* < 0.05) was observed between ^89^Zr-labeled ND (I) and ^68^Ga-labeled ND (III), both in SCID.

### 3.5. In Vivo Biodistribution Validation Based on PET and MRI Data

The highest activities were detected in liver and spleen, with the values for the ^89^Zr-labeled NDs in the same order of magnitude with respect to the activity concentration (Liver: (52.9 ± 12.9) %IA/mL in SCID mice (I, 168 h), (41.7 ± 7.2) in C57BL/6 mice (II, 24 h); Spleen: (42.0 ± 15.6) %IA/mL in SCID mice (I, 24 h), (52.5 ± 12.1) %IA/mL in C57BL/6 (II, 24 h)), while for the ^68^Ga-labeled particles clearly higher values were determined in the liver in comparison with the spleen (Liver: (54.6 ± 1.2) %IA/mL, Spleen (20.5 ± 9.4) %IA/mL (III, 3 h)).

The values for heart (blood pool) (1 h: (11.1 ± 5.5) %IA/mL (I), (16.1 ± 10.6) %IA/mL (II), (12.2 ± 6.7) %IA/mL (III)) and brain (1 h: (0.7 ± 0.4) %IA/mL (I), (1.0 ± 0.8) %IA/mL (II), (1.1 ± 0.7) %IA/mL (III)) were similar in each of the three models, and the decrease in signal over time was also observed for the PET data (Figure 5).

**Figure 7:**
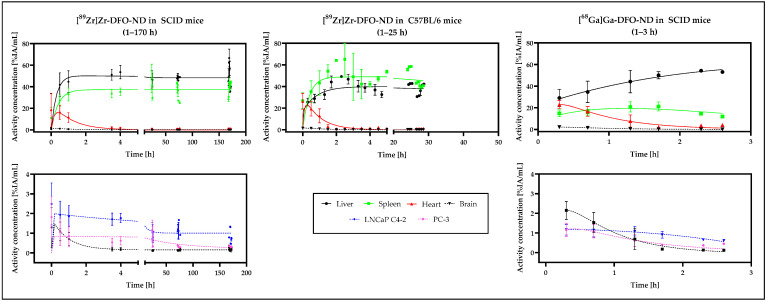
Time-activity curves (TACs) based on PET datasets were fitted using a three-exponential model in GraphPad Prism 9.4.0. TACs of liver, spleen, heart, and brain for the respective model (^89^Zr- and ^68^Ga-labeled NDs in SCID mice and ^89^Zr-labeled NDs in C57BL/6 mice, respectively) were visualized in the top row. In the bottom row, the TACs for the tumors (LNCaP C4-2 and PC-3) in the SCID mice were also presented, along with the brain data.

Based on the PET data, time–activity curves (TAC) for the volumes of interest (VOIs) were obtained, including liver, spleen, heart, brain, and the tumor regions (Figure 7).

A distinct increase in the signals (ND concentration) for spleen and liver was detected in the TACs from the time of injection to about 2 h after injection, whereas the corresponding signal in the heart area, after a short initial increase, continuously decreased. The same trend was observed for all three study models. Measurement after 3 h with ^89^Zr-labeled NDs revealed a slight decrease of the signals in the liver and spleen. The signal in the heart region remained low.

A similar overall weaker curve was found for the brain signal as for the heart.

Minor accumulation was detected in the tumors, which slowly decreased over time. The signal observed in the TCAs for LNCaP C4-2 was slightly higher than in PC-3.

For heart and brain, the time–activity curves of all three study models revealed a similar profile. In study models I and III (SCID mice), accumulation was higher in the liver than in the spleen, and vice versa in study model II (C57BL/6). Rapid accumulation and plateauing in liver and spleen was observed in all three study models. For both tumors, a similar curve, with decreasing activity concentration over time, was observed in Model I and III (SCID mice) as well. The activity concentration of PC-3 xenografts is similar in both models, whereas the activity concentration of LNCaP C4-2 in model I ([^89^Zr]Zr-DFO-ND in SCID) is higher than in model III ([^68^Ga]Ga-DFO-ND in SCID).

**Figure 8:**
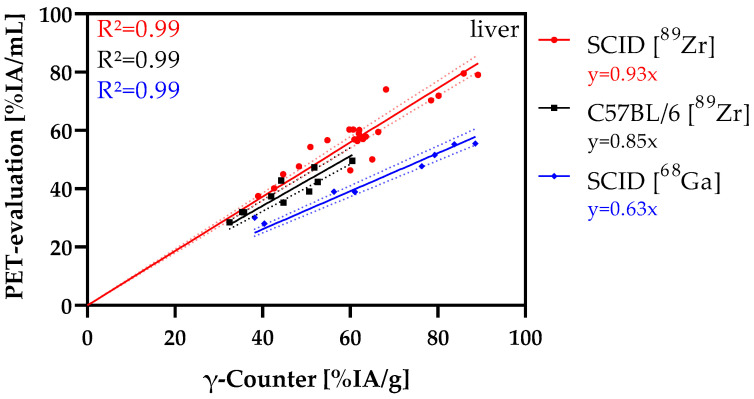
Comparison of PET-derived (ordinate) and gamma counter-derived (abscissa) liver data regarding the respective mouse model and radionuclide. The correlations between the two evaluation methods were additionally demonstrated by presenting the functional equations and the corresponding variances (R²).

Direct comparison of the liver data based on the gamma counter and PET data (Figure 8) demonstrated very good agreement of the results for the ^89^Zr-labeled NDs, both in SCID and in C57BL/6 mice. Variances (R^2^) close to 1 and slopes of the linear regression lines also close to y = x support these observations. For ^68^Ga-labeled NDs, the slope deviated more strongly from 1 (y = 0.63x) with a very good variance of 0.99.

The PET and gamma counter data for model I (SCID [^89^Zr]) and II (C57BL/6 [^89^Zr]) have good agreement with increases near the bisector with y = 1x. For model III (SCID [^68^Ga], there is a stronger deviation, indicating that the PET is underestimated compared to the gamma counter data. 

### 3.6. Correlation Analysis

The correlation of organ accumulation compared to the blood pool was analyzed. A linear correlation (Pearson) was determined for blood and brain (r = 0.988, *p* < 0.001), and for blood and small intestine (r = 0.811, *p* < 0.001) in study model I (^89^Zr-labeled NDs in SCID mice). PET data based on PMOD evaluation of the same model resulted in similar Pearson’s correlation of heart with LNCaP C4-2 (r = 0.625, *p* < 0.001), PC-3 (r = 0.582, *p* < 0.001), liver (r = −0.300, *p* < 0.001), spleen (r = −0.398, *p* < 0.001), and brain (r = 0.960, *p* < 0.001). 

For the combination of ^89^Zr-labeled NDs in C57BL/6 mice (study model II), Pearson’s correlations between the blood signal and brain (r = 0.941, *p* < 0.001), small intestine (r = 0.791, *p* = 0.006), muscle (r = 0.632, *p* = 0.05), and liver (r = −0.641, *p* = 0.05) were demonstrated based on gamma counter data. Analysis of PET data from ^89^Zr-labeled NDs in C57BL/6 mice resulted in Pearson correlation between heart and brain (0.962, <0.001), liver (−0.689, <0.001), and spleen (−0.340, <0.001).

Analysis of gamma counter data from ^68^Ga-labeled NDs in SCID mice (study model III) revealed Pearson correlations between blood and brain (r = 0.905, *p* = 0.002), lung (r = 0.833, *p* = 0.01), and liver (r = −0.794, *p* = 0.02). Based on PET analyses, Pearson correlations were obtained between heart and liver (r = −0.802, *p* < 0.001), spleen (r = −0.458, *p* < 0.001), and brain (r = 0.984, *p* < 0.001).

### 3.7. Tumor Uptake Analysis

**Figure 9:**
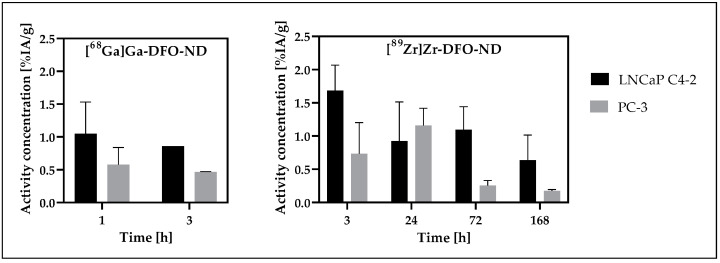
Tumor accumulation of radiolabeled NDs (%IA/g) in the prostate cancer tumor xenografts LNCaP C4-2 and PC-3. Mean and SD are depicted at the respective measurement time points based on gamma counter evaluation. Samples from n = 3 to n = 5 were available for each measurement time point, except [^68^Ga]Ga-DFO-ND (3 h): LNCaP C4-2 (n = 1), PC-3 (n = 2).

Tumor data were analyzed for initial evaluation of the NDs in the tumor-bearing mouse models (study models I and III). A low accumulation of radiolabeled NDs was observed for both tumor types, LNCaP C4-2 and PC-3 (Figure 9). Tumor analysis was performed for ^68^Ga-labeled NDs (III) at the early time points of 1 h and 3 h, while the later time points starting at 3 h were investigated with the long-lived radionuclide ^89^Zr (I). Overall, higher accumulation was obtained in the higher perfused tumor model LNCaP C4-2 for both radiolabeled NDs. Significance could not be tested for the ^68^Ga-labeled NDs due to the small sample size, while a significant difference (*p* < 0.05) between the two tumor types could be demonstrated for the ^89^Zr-labeled NDs over all measurement time points. For [^89^Zr]Zr-DFO-ND, a continuous decrease in accumulated activity was observed starting at the 3 h measurement point for LNCaP C4-2, and at 24 h for PC-3, respectively, after a temporary increase.

In both study models, higher accumulation was observed for the more perfused tumor xenograft LNCaP C4-2. With the exception of the 24 h time point, where a high variance for LNCaP C4-2 occurred, significant differences in uptake between tumor entities at the various time points could be demonstrated for study model I ([^89^Zr]Zr-DFO-ND in SCID).

**Figure 10:**
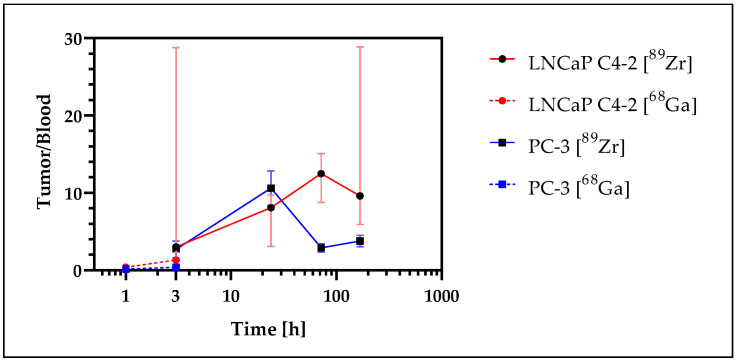
Combined graphs of tumor-to-blood ratios (median and interquartile range) of the [^68^Ga]Ga-DFO-ND (1 h, 3 h) and [^89^Zr]Zr-DFO-ND (3 h, 24 h, 72 h, 168 h). The time axis was scaled in logarithmic order.

To evaluate tumor-to-blood ratios, data from the ^68^Ga- and ^89^Zr-labeled NDs were combined in one graph (Figure 10). The ratio continuously increased over 24 h for both xenograft models. The values decreased significantly for PC-3 after 24 h, while for LNCaP C4-2 a further increase to the 72-h measurement was observed before the values decreased. Maximum median values of 10.4 (PC-3) and 12.5 (LNCaP C4-2) were determined. The results for ^68^Ga and ^89^Zr were in a good agreement. The ratio data were summarized in Table S4.

At the early time points of 1 h and 3 h p.i., a slight increase in tumor-to-blood ratio was observed in both xenograft models, and the accumulated activity at 3 h was in the same range for both study models ([^89^Zr]Zr-DFO-ND in SCID mice (I) and [^68^Ga]Ga-DFO-ND in SCID mice (III)). A similar trend was observed for both tumor models up to 24 h p.i., while for later time points a higher uptake was obtained in the LNCaP C4-2 tumor compared to PC-3.

## 4. Discussion

Radiolabeling and analysis of the biodistribution of NDs by PET and MR imaging in different mouse models over different time scales using short- (^68^Ga) and long-lived (^89^Zr) radionuclides were successfully performed, thus providing a generic non-invasive imaging platform for ND pharmacokinetic assessment. It was demonstrated that the immunodeficient as well as immunocompetent mouse strains are appropriate for both distribution and accumulation studies. Radiolabeled NDs behaved comparable to what is known from other radiolabeled NPs with accumulation predominantly in the RES.

### 4.1. Radiolabeling of NDs

Radiolabeling of HSA-coated NDs with the long-lived radionuclide ^89^Zr was successfully performed, resulting in a high radiochemical yield and very good stability. The radiolabeling of the NDs was remarkably efficient, which is in good compliance to ^89^Zr-labeling reported for other nanoparticles [29,59,60,61]. As an alternative to ^89^Zr labeling, labeling with ^68^Ga was slightly less efficient but still provided sufficient radiochemical purity. Similar labeling efficiency was also reported by Baur et al. [32].

With regard to the biodistribution and in vivo stability of DFO coupled compounds, Petrik et al. [58] reported similar characteristics for ^68^Ga and ^89^Zr. Further, coating stability was demonstrated by Moscariello et al. [37] for in vivo blood–brain barrier transport. In the case of free ^68^Ga and ^89^Zr, previous studies reported almost uniform biodistribution [29,62]. In our study, we did observe organ-specific accumulation, thus indicating stable binding of the radionuclides to the NDs in vivo.

### 4.2. Biodistribution of Nanodiamonds in Normal Organs

For all mouse models, primary accumulation was detected in the organs of the reticuloendothelial system, specifically the liver and spleen in this case. According to determined activity concentrations based on both PET and gamma counter measurements, as well as the correlation analyses in comparison with the blood concentration, the accumulation in the filtration organs was confirmed. The same methods were used to correlate the minor accumulation in the brain, small intestine, and muscle with the concentration of nanodiamonds in the blood.

No substantial differences in organ biodistribution of nanodiamonds were observed between the two mouse models used in this study. Variances in liver and spleen uptake could be attributed to phenotypic differences between the mouse models.

A comparison of the PET and gamma counter data revealed excellent correlation of the zirconium-89 data in both SCID and C57BL/6 mice. Therefore, both methods lead to the same conclusions based on the data obtained. For the ^68^Ga—model (III), the correlations were still good, but the data were shifted to higher gamma counter values. In comparison with the other two models (I, II), the difference was caused by the radionuclide used, since the SCID mice were also used with ^89^Zr. A major difference between ^68^Ga and ^89^Zr in terms of PET measurements and quantification is the positron range (PR), the resulting influence on the spatial resolution, and thus also on the partial volume effect (PVE) [63,64]. The positron range of ^68^Ga (0.337 cm) is 2.7-fold larger than for ^89^Zr (0.123 cm) and thus has a significant influence on the overall resolution.

For the biodistribution of these non-functionalized NDs, the expected pattern of rapid uptake into the organs of the reticuloendothelial system and limited circulation time with accumulation in the liver and spleen already for the 1 h measurement were observed. The “trapping” of NDs in the respective organs is documented by the largely stable concentrations observed at time points up to 7 d after injection and by the low radiation observed in other organs and tissue samples. These results correspond to biodistribution studies with ND published before, where accumulation for long periods of time was also observed primary in liver, spleen, and lung [21,24,25]. The low accumulation of the studied NDs in the lungs compared with published data may indicate a beneficial effect of HSA coating.

Studies with the ND used as well as with similar particles could not detect toxicity, even over longer periods of time [23,24,34]. Over the measurement period of seven days, the mice in our approach also showed no health abnormalities, supporting the assumption that no toxic effect was induced by ND accumulation.

Lack of signal accumulation in kidney and bladder or in bones and joints is a clear indication of the good radiolabeling stability, as free ^89^Zr is osteotropic and excreted via the kidneys [29,65]. Concentration variations in spleen in organ-weight-normalized analysis is attributed to an offset problem of the balance used for weight measurements. The PET data are thus suitable for quantitative evaluation of the biodistribution and accumulation of ^89^Zr-labeled NDs, whereas for ^68^Ga-labeled NDs significantly lower values were detected due to the PVE. For ^68^Ga, problems arise for measurements in small animals, especially for smaller organ structures, necessitating partial volume correction (PVC).

### 4.3. Accumulation of NDs in Tumor Xenografts

The accumulation in the tumor xenografts correlated with the known perfusion of the respective xenograft, indicating that the accumulation was predominantly caused by the EPR effect. Successful transport of NDs into the tumor xenografts is clearly demonstrated by the tumor-to-blood ratios with values > 1. The decrease in tumor-to-blood ratio after 168 h likely indicates nanoparticle decomposing, which might even be favorable with respect to ND excretion. 

In studies with PEG- and doxorubicin-coupled NDs by Madamsetty et al. [23], an improved therapeutic effect was observed compared to doxorubicin alone. Here, the nonspecific uptake of the PEG-modified NDs by the EPR effect appeared to increase doxorubicin concentration in the tumor. At the same time, accumulation in organs of the RES and toxic effects were low. The PEG modification seemed to cause an improved masking of these NDs. The analysis of biodistribution based on ND fluorescence could only be performed semiquantitatively.

For the NDs investigated, a combination of PEG and HSA coating was used, which needs further optimization. However, the corresponding evaluation of a modified coating can be performed with the presented platform that allows for absolute quantification.

The presented data nicely demonstrate that the pharmacokinetics of the NDs can be accurately assessed by the PET labelling approach. Even though the rather slow uptake of the NDs into the tumors may not be limiting for drug carriage, it indicates a general limitation of using hyperpolarized NDs as imaging agents. Where the hyperpolarization in NV bulk diamonds shows long half-life times in the multiple hour range, in NDs the hyperpolarization is still limited to the minute range. For usage as an imaging agent, either further functionalization, ensuring more rapid uptake of the NDs in the tumor, or surface modified NDs showing longer half-life need to be investigated.

### 4.4. Variance in Mouse Models

The mean body weight of the mice tested was nearly identical for both strains. No differences in organ weights were detected for the lungs, heart, kidneys, and brain. However, for liver and spleen, significant differences in organ weights were observed between the C57BL/6 and SCID mouse strain. The weight differences remained the same over the range of measurement, thus indicating characteristics of the animal models and cannot be attributed to the nanodiamonds.

Liver and spleen, as part of the reticuloendothelial system also play a crucial role for the immune system [66,67,68,69,70,71,72,73]. Differences between immunodeficient and wild-type mouse models with respect to the spleen have been previously published based on MR and histopathological studies [74,75].

For the SCID model, it is known that thymus and lymph nodes are abnormally small and are devoid of lymphocytes, which is also true for splenic follicles (Charles River Technical sheet; The Jackson Laboratory). The structural organ differences may not only result in functional differences of the immune system, but also potentially have effects on the function as a filtering organ of the RES.

However, despite the documented differences, the use of SCID mice in our studies provides results similar to the C57BL/6 mouse model in terms of biodistribution.

### 4.5. Limitations

Up to now, no direct comparison with results from MR with analogous hyperpolarized substances was performed. For future comparison to hyperpolarized nanodiamonds, possible effects of the surface modification on the biodistribution should be considered. For optimum comparability, identical surface modifications should also be applied to the hyperpolarized NDs. The non-radioactive isotopes of the radionuclides, zirconium or gallium, can be added in the appropriate concentrations, resulting in chemically identical however non-radioactive compounds.

Additional information can be obtained by histological preparation of accumulating organs and tumors for toxicological analyses. Furthermore, immunohistochemical detection of HSA by antibody labeling could help to analyze the distribution within the tumor or even the organs of the RES (liver, spleen, lung). Electron microscopic analyses of the HSA-positive regions would subsequently provide information on the stability of the coating.

## 5. Conclusions

The results demonstrate the applicability of radiolabeled NDs to assess the long- and short-term biodistribution of NDs after systemic injection. This experimental platform can be used in future studies to characterize and quantify the targeting properties of NDs additionally functionalized for specific tumor types and serve as a reference for future MR studies with hyperpolarized NDs.

## Figures and Tables

**Table 1:** Overview of the experiments performed with details of the respective radionuclide, number of animals, and injected mass [µg] and injected activity ([MBq], mean and standard deviation (SD)).

Study Model	I	II	III
Radionuclide	^89^Zr	^89^Zr	^68^Ga
Mouse strain	CB17/lcr-Prkdc scid/Crl	C57BL/6NCrl	CB17/lcr-Prkdc scid/Crl
Number of animals (n=)	25 *	10	8 ^†^
Time Points	1 h, 3 h, 24 h, 3 d, 7 d	1 h, 3 h, 24 h	1 h, 3 h
Injected activity [MBq] (avg. ± SD)	1.41 ± 0.42	1.46 ± 0.39	6.08 ± 1.53
ND [µg]	40.4 ± 7.7	43.5 ± 4.8	34.7 ± 9.5

* subset of n = 15 with tumor xenografts; ^†^ with tumor xenografts.

## Data Availability

The used data, additional to those in the supplement, are available from the corresponding author on reasonable request.

## Supplementary Materials

The following are available online at https://www.mdpi.com/article/10.3390/nano12244471/s1, Figure S1: Maldi-TOF spectra of cHSA, cHSA-PEG, and cHSA-PEG-DFO. Figure S2: TEM image of nanodiamonds (S2A), as well as DLS spectra of uncoated and coated nanodiamonds (S2B). Figure S3: Schematic overview of the radiolabeling procedure. Figure S4: Schematic overview to visualize the imaging experiments procedure. Figure S5: PET-images of [^68^Ga]Ga-DFO-ND biodistribution in mice. Figure S6: Comparison of activity concentration [%IA/g] in liver and spleen between the models at the monitored time points based on gamma-counter data. Figure S7: Comparison of relative accumulated activity [%IA] in liver and spleen between the models at the monitored time points based on gamma-counter data. Table S1: Mean and standard deviation [%IA/g] of the biodistribution of [^89^Zr]Zr-DFO-ND in SCID mice. Table S2: Mean and standard deviation [%IA/g] of the biodistribution of [^89^Zr]Zr-DFO-ND in C57BL/6 mice. Table S3: Mean and standard deviation [%IA/g] of the biodistribution of [^68^Ga]Ga-DFO-ND in SCID mice. Table S4: Tumor-to-blood ratios and tumor ratios (PSMA^+^-to-PSMA^−^).
