# Supplementary material for: Preclinical PET and MR Evaluation of 89Zr- and 68Ga-Labeled Nanodiamonds in Mice over Different Time Scales
Source: Nanomaterials (Basel). 2022 Dec 16;12(24):4471. doi: 10.3390/nano12244471 (PMC9780863; doi:10.3390/nano12244471)
Supplement: Supplementary file 1 [file nanomaterials-12-04471-s001.zip › nanomaterials-2064147-supplementary.pdf]

nanomaterials

MDPI

Supplement

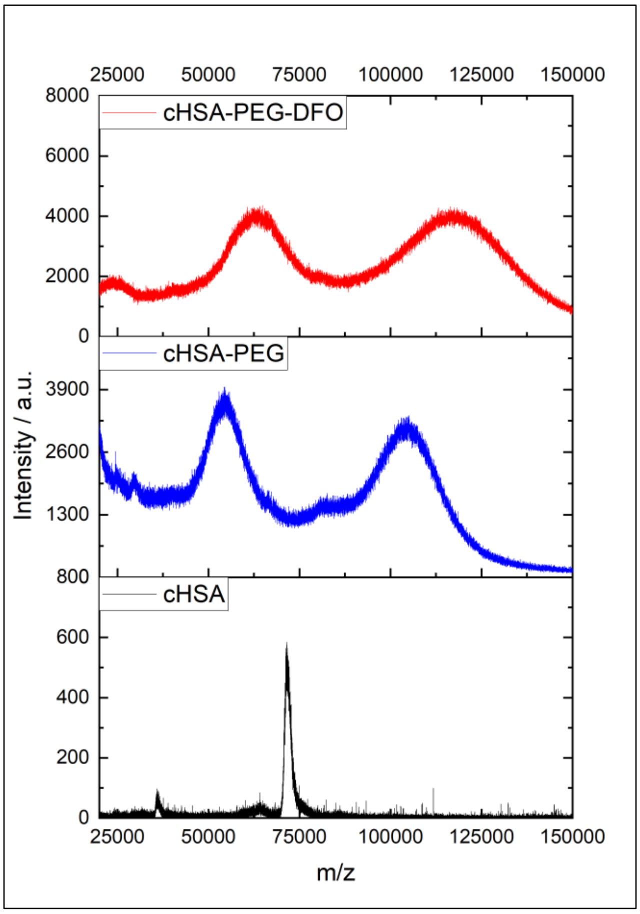

Figure S1: Maldi-TOF spectra of cHSA (black, 72 kDa), cHSA-PEG (blue, 105 kDa), and cHSA-PEG-DFO (red, 116 kDa).

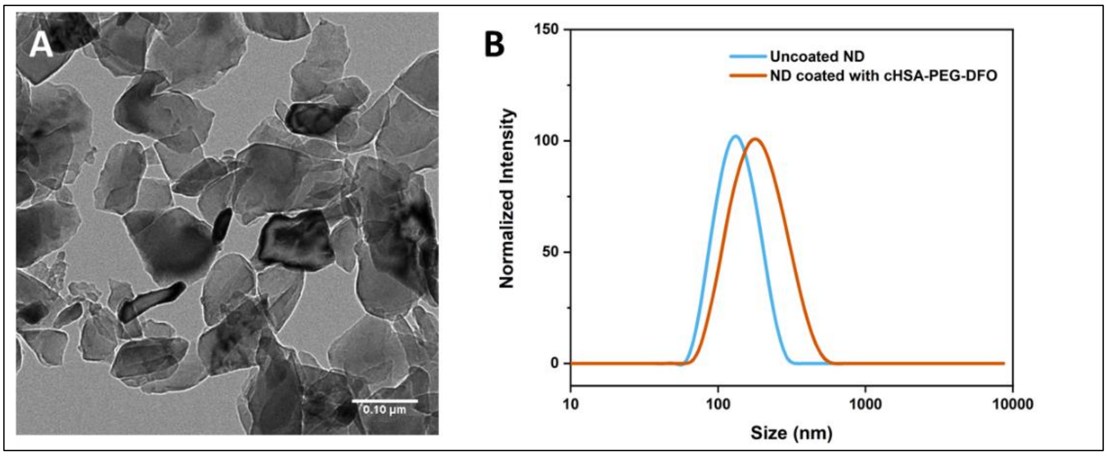

Figure S2: Transmission electron microscopy (TEM) of uncoated NDs indicating an average size of about 100 nm in diameter (A). Dynamic light scattering of uncoated NDs (B, blue, diameter: 130.8 ± 2.9 nm, PDI: 0.086) and NDs coated with cHSA-PEG-DFO (B, orange, diameter: 170.1 ± 0.9 nm, PDI: 0.133).

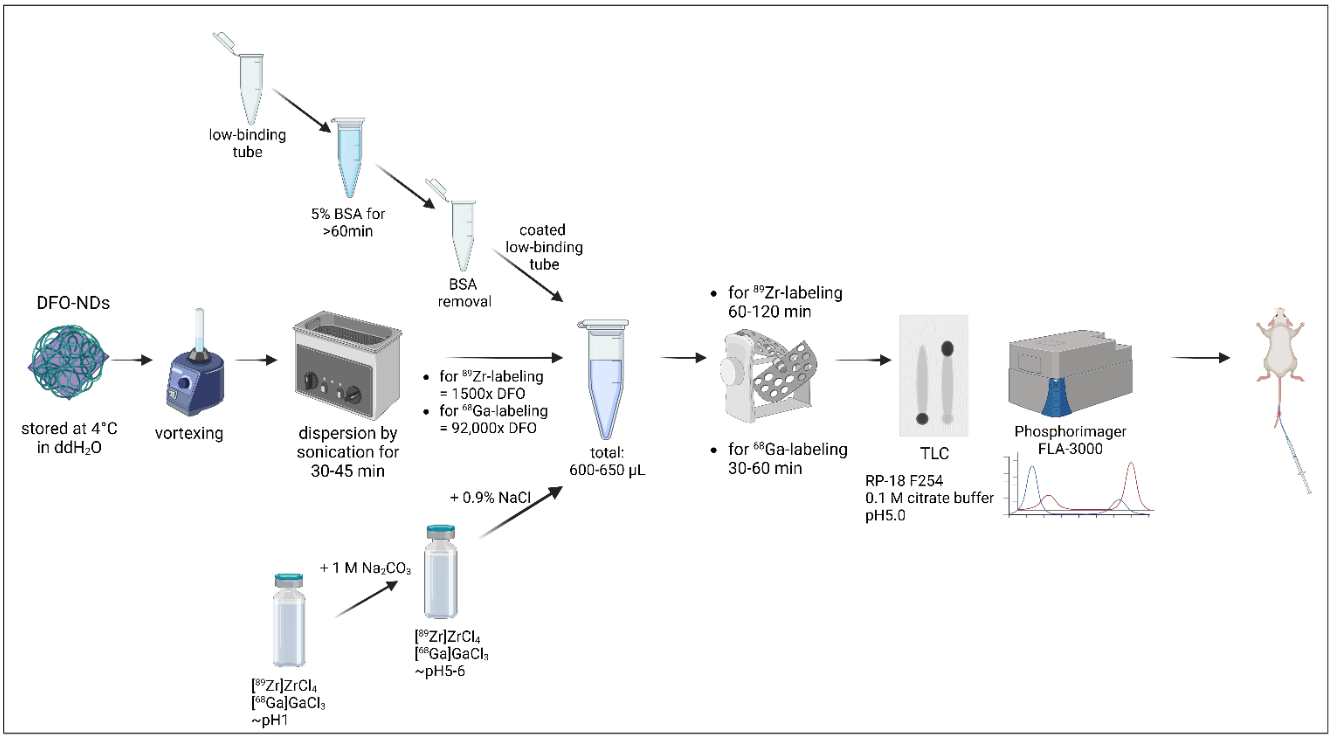

Figure S3: Schematic overview of the radiolabeling procedure. Nanodiamonds were prepared as described and provided in double distilled water. Transport and storage occurred at 4°C. For application, the diamonds were carefully vortexed and subsequently dispersed by ultrasonication. In parallel, zirconium-89 or gallium-68 were prepared and adjusted to a pH of 5-6, and a reaction vial was coated with BSA to reduce wall effects. In a total volume of 600-650 µl, the batches were incubated in an overhead shaker, in time according to the radionuclide used. Successful radiolabeling was subsequently verified by thin layer chromatography and autoradiography. After the tests, the solution was ready for injection. (Created with BioRender.com)

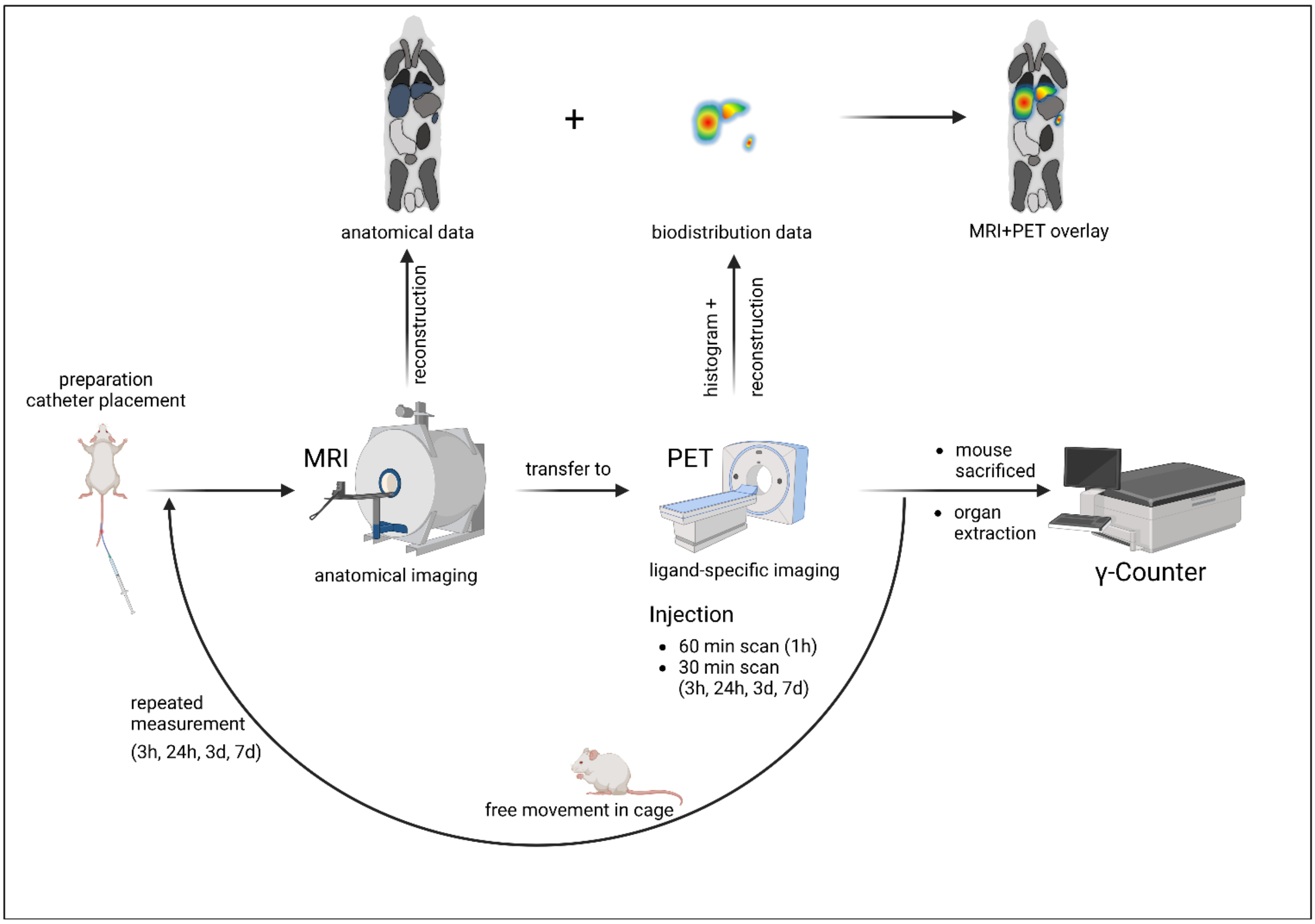

Figure S4: Schematic overview to visualize the imaging experiment procedure. In preparation, the catheter was placed, and the mouse was positioned in the bed for MRI and PET measurements. MRI and PET measurements were performed according to the protocols in the method section. The first dynamic PET measurement was always performed over a measurement period of 1 h to monitor the distribution after injection of the radioligand. Repetitive measurements at later time points were also performed dynamically for at least 30 min. For each time point, mice were sacrificed to remove the organs and quantify the accumulated radioactivity in the γ-counter. The acquired MRI and PET data were reconstructed and overlaid to allow better localization of the signal. (Created with BioRender.com)

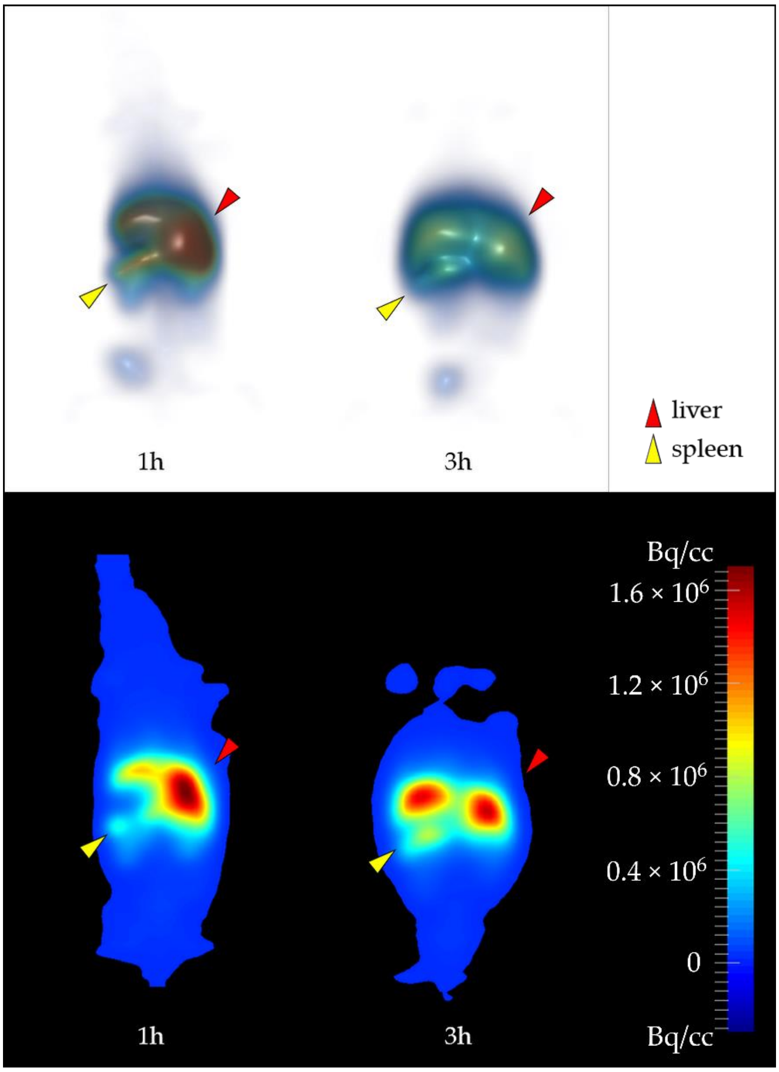

Figure S5: PET-images of [^{68}Ga]Ga-DFO-ND biodistribution in mice. Visualized in the example figures is the biodistribution of the ^{68}Ga-labeled nanodiamonds based on PET imaging. Both mice were administered 56.3 µg of radiolabeled NDs with a radioactivity of 5.7 MBq (1 h) and 5.6 MBq (3 h). In the upper row a maximum intensity projection (MIP) in posterior view was depicted and the visible organs were marked by arrows. Additionally, coronal 2D PET images in posterior view were added. The labeled organs are Li=liver, Sp=Spleen, Bl=bladder, and K=Kidney areas. Both illustrations were based on identical scaling settings.

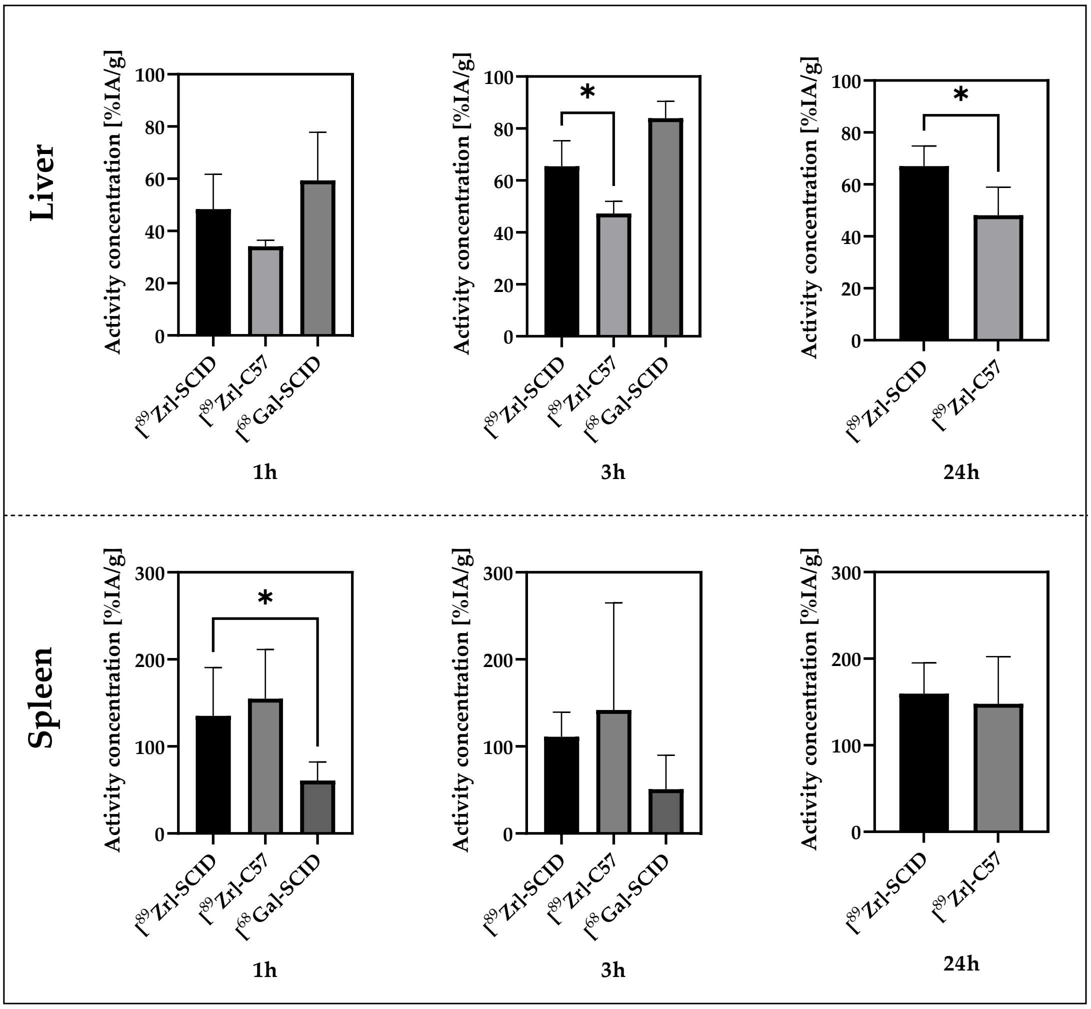

Figure S6: Comparison of activity concentration [%IA/g] in liver and spleen between the models at the monitored time points based on gamma-counter data. Mann-Whitney test was performed to check for significance between [^{89}Zr]Zr-DFO-ND in SCID mice and in C57BL/6 mice as well as between [^{89}Zr]Zr-DFO-ND and [^{68}Ga]Ga-DFO-ND in SCID mice. For ^{68}Ga-labeled NDs data were only available at 1 h and 3 h. Significant difference (*, $p \leq 0.05$) was observed for liver at 3 h and 24 h between the different mouse strains and for spleen at 1 h for ^{89}Zr- and ^{68}Ga-labeled NDs in SCID mice.

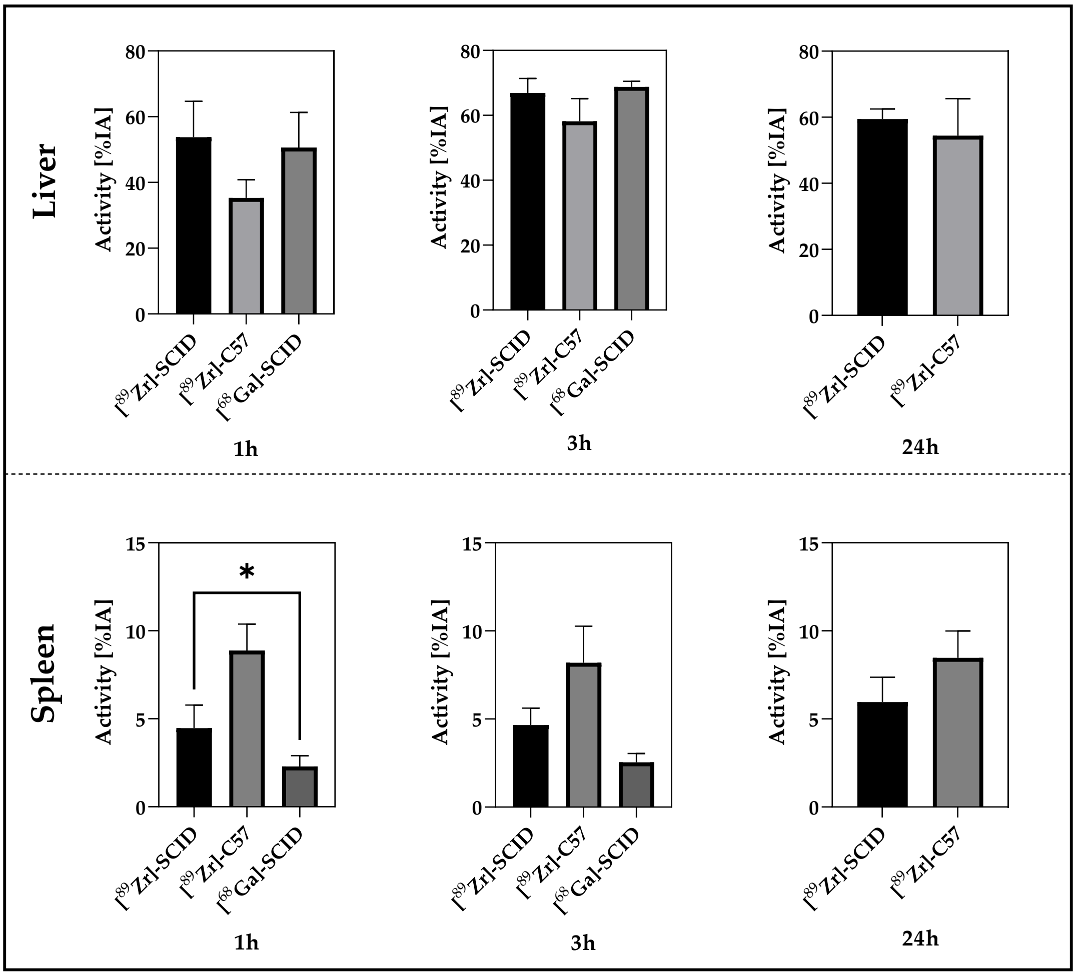

Figure S7: Comparison of relative accumulated activity [%IA] in liver and spleen between the models at the monitored time points based on gamma-counter data. Organ weight is disregarded since complete organs were extracted. Mann-Whitney test was performed to check for significance between [^{89}Zr]Zr-DFO-ND in SCID mice and in C57BL/6 mice as well as between [^{89}Zr]Zr-DFO-ND and [^{68}Ga]Ga-DFO-ND in SCID mice. For ^{68}Ga-labeled NDs data were only available at 1 h and 3 h. Significant difference (*, $p \leq 0.05$) was observed for spleen at 1 h for ^{89}Zr- and ^{68}Ga-labeled NDs in SCID mice.

Table S1: Mean and standard deviation [%IA/g] of the biodistribution of [^{89}Zr]Zr-DFO-ND in SCID mice. For all mice, biodistribution was evaluated (n=25) while tumor data were based on a subset of these mice with tumor xenografts established (n=15).

Time-point	Blood	Heart	Brain	Lung	Liver	Spleen	Kidney	Colon	Small Intestine	Muscle	Bone	LNCaP C4-2	PC-3
**1**	4.84 ± 4.30	4.61 ± 4.18	0.161 ± 0.108	5.11 ± 1.98	48.39 ± 13.29	135.09 ± 55.48	5.51 ± 2.85	2.12 ± 2.72	3.69 ± 3.45	0.32 ± 0.27	2.52 ± 1.19	-	-
**3**	0.34 ± 0.26	1.34 ± 0.31	0.052 ± 0.011	2.31 ± 0.93	65.50 ± 9.83	111.00 ± 28.48	6.47 ± 2.16	1.11 ± 1.04	2.21 ± 1.75	0.12 ± 0.04	2.68 ± 1.07	1.68 ± 0.39	0.73 ± 0.47
**24**	0.12 ± 0.05	0.44 ± 0.04	0.039 ± 0.005	2.66 ± 1.02	67.06 ± 7.72	159.55 ± 35.56	7.28 ± 2.38	0.98 ± 0.62	1.51 ± 0.93	0.09 ± 0.04	3.28 ± 0.58	0.93 ± 0.59	1.16 ± 0.26
**72**	0.08 ± 0.02	0.93 ± 0.51	0.033 ± 0.002	1.18 ± 0.12	61.36 ± 0.64	100.66 ± 43.67	2.80 ± 0.28	0.34 ± 0.04	1.38 ± 0.29	0.09 ± 0.03	2.06 ± 0.28	1.09 ± 0.35	0.25 ± 0.07
**168**	0.05 ± 0.02	1.43 ± 1.63	0.036 ± 0.003	1.45 ± 0.50	65.36 ± 17.47	180.40 ± 147.97	3.44 ± 0.65	0.29 ± 0.19	0.98 ± 0.62	0.08 ± 0.03	2.19 ± 0.35	0.64 ± 0.38	0.18 ± 0.02

Table S2: Mean and standard deviation [%IA/g] of the biodistribution of [^{89}Zr]Zr-DFO-ND in C57BL/6 mice (n=10).

Time-point	Blood	Heart	Brain	Lung	Liver	Spleen	Kidney	Colon	Small Intestine	Muscle	Bone
**1**	17.84 ± 2.40	3.86 ± 0.12	0.46 ± 0.12	5.91 ± 1.78	34.13 ± 2.31	154.97 ± 56.48	4.07 ± 0.10	0.63 ± 0.08	13.28 ± 6.13	0.37 ± 0.02	2.34 ± 0.28
**3**	1.51 ± 2.06	2.76 ±2.35	0.12 ±0.08	2.09 ± 0.30	47.31 ± 4.67	141.84 ± 122.99	3.84 ± 0.45	0.52 ± 0.12	2.35 ± 1.09	0.24 ± 0.19	3.29 ± 2.05
**24**	0.12 ± 0.02	1.12 ±0.63	0.04 ± 0.01	1.29 ± 0.38	48.13 ± 10.83	147.84 ± 54.51	2.77 ± 0.65	0.56 ± 0.18	5.61 ± 2.72	0.16 ± 0.06	3.32 ± 2.21

Table S3: Mean and standard deviation [%IA/g] of the biodistribution of [^{68}Ga]Ga-DFO-ND in SCID mice (n=8).

Time-point	Blood	Heart	Brain	Lung	Liver	Spleen	Kidney	Colon	Small Intestine	Muscle	Bone	LNCaP C4-2	PC-3
**1**	11.61 ± 7.41	2.32 ± 1.34	0.30 ± 0.21	5.37 ± 1.37	59.36 ± 18.46	60.83 ± 21.27	9.95 ± 5.50	0.60 ± 0.18	5.40 ± 6.08	0.31 ± 0.18	1.53 ± 0.46	1.05 ± 0.48	0.58 ± 0.26
**3**	0.74 ± 0.11	0.283 ± 0.005	0.04 ± 0.02	1.61 ± 0.07	83.95 ± 6.55	50.84 ± 39.06	6.28 ± 0.54	0.65 ± 0.53	1.42 ± 0.03	0.24 ± 0.11	0.72 ± 0.71	0.86 (n=1)	0.47 ± 0.01

Table S4: Tumor-to-blood ratios and tumor ratios ($PSMA^+$-to-$PSMA^-$). The tables were based on the data from biodistribution analysis in SCID mice using [^{89}Zr]Zr-DFO-ND for up to 168 h p.i. (n=15) and using [^{68}Ga]Ga-DFO-ND (n=8) for up to 3 h.

**Time-point**	**Tumor/Blood LNCaP C4-2**	**Tumor/Blood PC-3**	**LNCaP C4-2 / PC-3**
**1**	-	-	-
**3**	11.37 ± 15.09	2.71 ± 1.85	3.22 ± 2.59
**24**	6.97 ± 3.73	10.62 ± 4.48	0.83 ± 0.54
**72**	12.11 ± 3.46	2.89 ± 1.09	4.82 ± 2.92
**168**	14.80 ± 13.38	3.78 ± 1.52	3.54 ± 1.79

**Time-point**	**Tumor/Blood LNCaP C4-2**	**Tumor/Blood PC-3**	**LNCaP C4-2 / PC-3**
**1**	0.38 ± 0.68	0.15 ± 0.23	2.61 ± 1.27
**3**	1.30*	0.64 ± 0.09	1.86*

***n=1**
